# Fibrofolliculomas in Birt-Hogg-Dubé syndrome treated with nonfractionated ablative CO_2_ laser

**DOI:** 10.1016/j.jdcr.2023.08.026

**Published:** 2023-08-30

**Authors:** Rishi Patel, Jesse Wesenberg, Jennifer Brammeier

**Affiliations:** a College of Osteopathic Medicine, Lake Erie College of Osteopathic Medicine, Bradenton, Florida; b Arsenault Dermatology, Department of Dermatology & Mohs Micrographic Surgery, Lakewood Ranch, Florida

**Keywords:** ablative laser, Birt-Hogg-Dubé, CO_2_ laser, fibrofolliculoma, fractionally ablative laser, nonfractionated laser, trichodiscoma

## Introduction

Birt-Hogg-Dubé syndrome (BHDS) is a rare autosomal dominant genodermatosis associated with a mutation in the folliculin (*FLCN*) gene resulting in benign cutaneous lesions and ominous systemic manifestations, including pneumothorax and renal carcinoma.^1^ Cutaneous manifestations of BHDS are benign and include trichodiscomas, perifollicular fibromas, acrochordons, and numerous fibrofolliculomas associated with the hair follicles on the face, ears, head, neck, and chest.^2^ Despite the benign nature, the texture and appearance of hundreds of coalescing fibrofolliculomas and benign growths can have an important psychologic effect and be cosmetically unsatisfactory to patients with BHDS. We report successful treatment of fibrofolliculomas in a patient with BHDS using a combination of fractionated and nonfractionated ablative CO_2_ 10,600 nm laser (CO2RE, Candela) resulting in improvement of the lesion count, lesion size, and overall textural appearance.

## Case report

**Fig 1:**
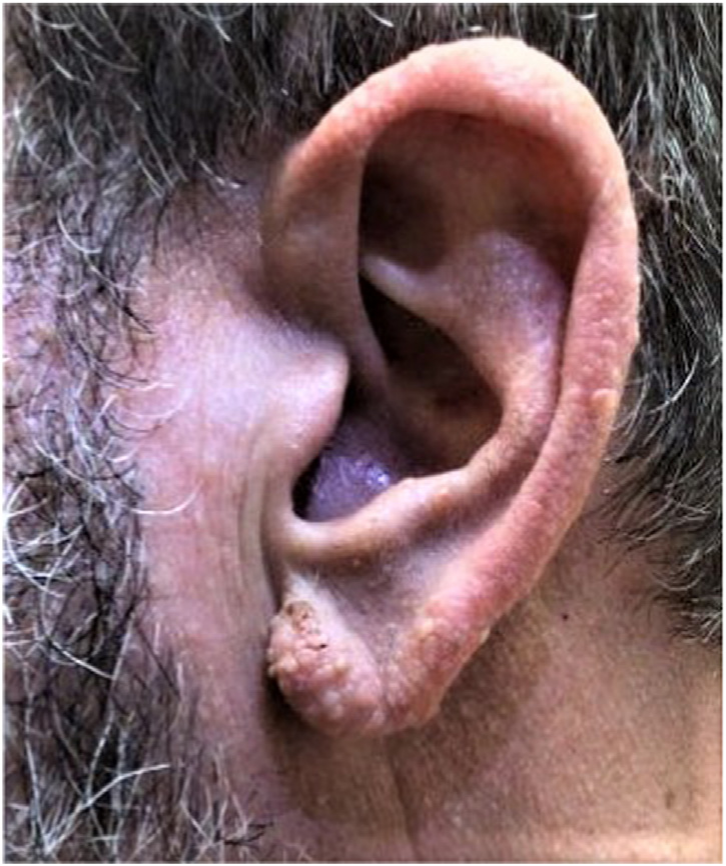
Numerous fibrofolliculomas distributed along the ear prior to treatment.

**Fig 2:**
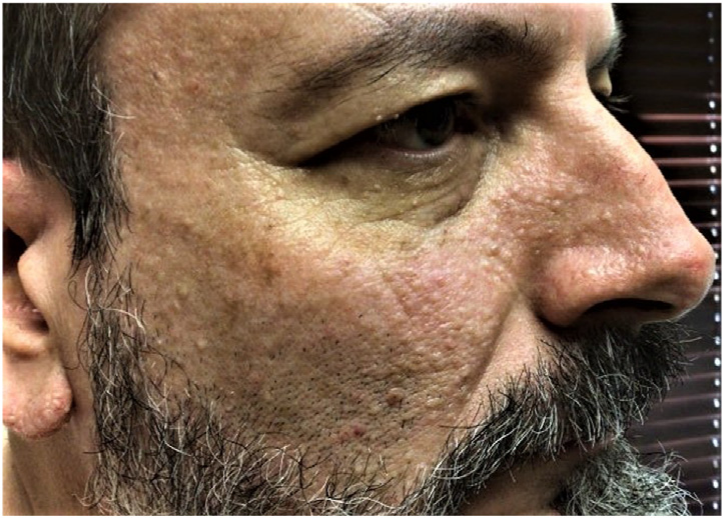
Numerous fibrofolliculomas distributed throughout the right malar cheek and nose before treatment.

**Fig 3:**
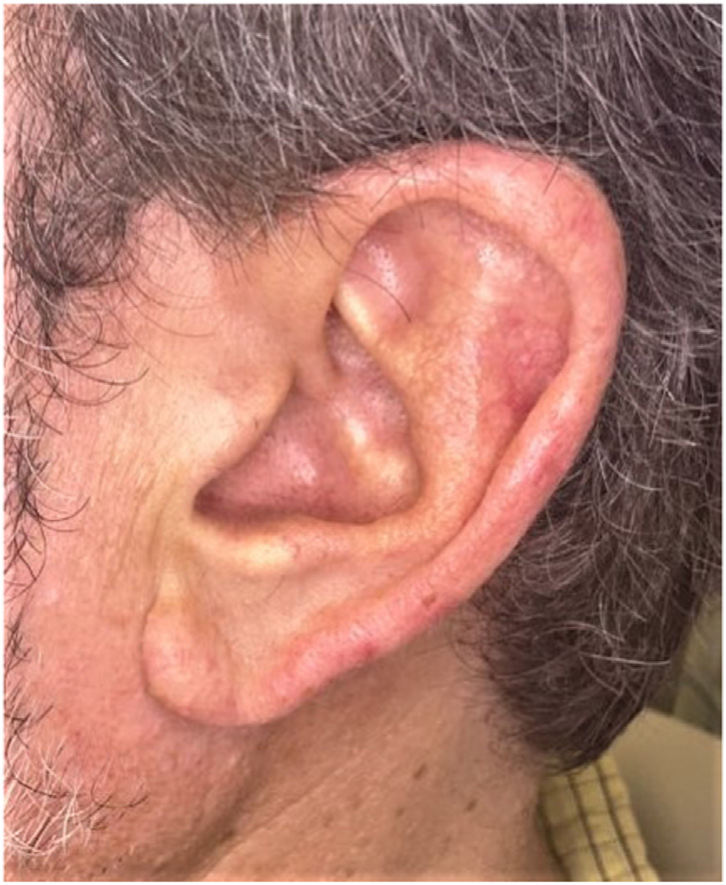
Smooth texture and contour of the ear after CO_2_ laser.

**Fig 4:**
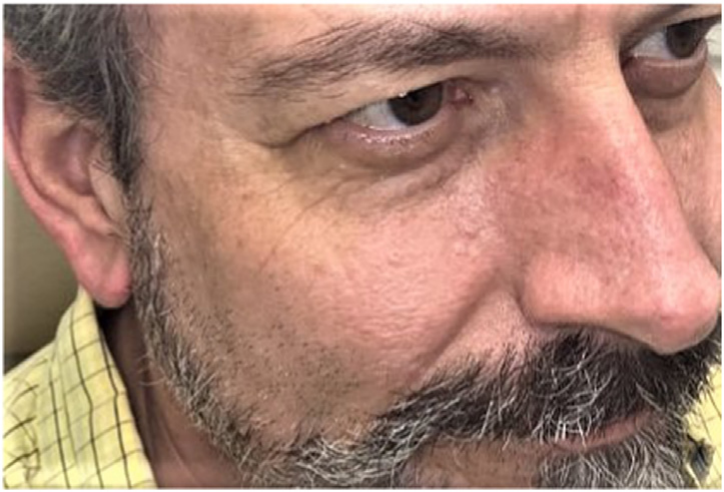
Improved texture and lesion count on the right malar cheek and nose after treatment with CO_2_ laser.

A 55-year-old Caucasian man with a history of known BHDS presented seeking treatment for numerous firm flesh-colored papules diagnosed as fibrofolliculomas distributed on the face and bilateral ears. Facial lesions were primarily concentrated on the bilateral malar cheeks and nose, with fewer lesions on the forehead, in the periorbital region, and within the beard distribution (Figs 1 and 2). The initial lesion count on the face and ears was 256, excluding the beard distribution. The patient was counseled on the potential benefits and adverse effects of CO_2_ laser resurfacing and was provided detailed preoperative and postoperative instructions. The patient was prescribed prophylactic valacyclovir 500 mg, which was initiated the day prior to his procedure, and cephalexin 2 g, which was initiated 1 hour before the procedure. Supraorbital, infraorbital, and mental nerve blocks were performed using lidocaine, and topical benzocaine 20%–lidocaine 10%–tetracaine 4% was applied for anesthesia. The first laser session consisted of conservative therapy using a combination of nonfractionated ablative and fractionally ablative CO_2_ settings. Distinct lesions were first treated with the nonfractionated ablative classic mode (3 mJ); then, the entire face and ears were resurfaced using the fractionally ablative setting mid mode (30% fractional coverage, ring size 3, ring 171 mJ). At his 1-month follow-up, the patient had tolerated the procedure well with no side effects and noted improvement in texture, but distinct lesions remained identifiable. Two months later, a second, more aggressive laser session was performed. Distinct lesions were first treated with the nonfractionated ablative classic mode (6 mJ); then, the entire face and ears were resurfaced using the nonfractionated ablative classic mode (3 mJ). At his 1-month follow-up, there was significant improvement in lesion count, lesion size, and overall texture. There was a 92% reduction in the lesion count, with an initial count of 256 and a final count of 21. Diffuse erythema and a mild pustular acneiform eruption were noted upon examination and resolved with 1 session of pulse dye laser (595nm) and oral doxycycline 100 mg twice daily for 2 weeks, respectively. At his 3-month follow-up, the patient was satisfied with the results and demonstrated marked sustained improvement in lesion number, size, and texture (Figs 3 and 4). At his 6-month follow-up, there was minimal recurrence and 7 individual refractory fibrofolliculomas were treated with the spot treatment nonfractionated ablative CO_2_ classic mode (6 mJ).

## Discussion

BHDS is a genodermatosis caused by a mutation in the *FLCN* gene located on chromosome 17p11.2 that results in numerous benign cutaneous growths, pulmonary cysts, pneumothorax, and renal tumors, including renal cell carcinoma.^1^ Benign cutaneous lesions seen in BHDS include trichodiscomas, perifollicular fibromas, acrochordons, and most commonly fibrofolliculomas.^2^ The diagnosis of BHDS is based on recognition of at least 5 cutaneous fibrofolliculomas and/or trichodiscomas, with histologic confirmation of at least 1 of the lesions.^1^ Fibrofolliculomas are folliculosebaceous hamartomas that develop in 75% to 90% of Caucasian patients with BHDS.^1^^,^^3^ Although fibrofolliculomas are benign, they can have an important psychologic effect and can cause cosmetic dissatisfaction in patients with BHDS.

Fibrofolliculomas can be refractory to or recurrent after treatment. Treatment options for fibrofolliculomas include electrocautery, shave removal, dermabrasion, excision, topical rapamycin, and ablative lasers.^4^, ^5^, ^6^, ^7^, ^8^ CO_2_ lasers emit energy at a wavelength of 10,600nm, which is selectively absorbed by water within the skin, causing vaporization, which makes this laser excellent for cutting, coagulation, and ablation of the skin.^9^ CO_2_ laser and erbium:yttrium-aluminum-garnet ablative lasers have been reported as effective and fast options for removal of numerous lesions with few side effects and without adverse effects.^4^^,^^6^, ^7^, ^8^ Truchuelo et al^4^ reported 2 cases treated with ablative CO_2_ laser resulting in 75% clearance of facial lesions, with the maintenance of clearance sustained after 18 months and 4 years. A case study conducted by Gambichler et al^6^ reported that erbium:yttrium-aluminum-garnet was useful because it can treat lesions that are traditionally difficult to treat, such as those on ears; however, lesions recurred after 6 months, possibly due to the depth of penetration not reaching far enough within the dermis. Consistent with these previous findings, we report a similar case with 92% reduction in lesion count after 2 ablative CO_2_ sessions. Our patient experienced prolonged erythema for 6 weeks and mild pustular acneiform eruption, which responded well to pulse dye laser and short-term oral antibiotic therapy. Adverse effects of ablative lasers include persistent erythema, hyperpigmentation, hypopigmentation, infections, scarring, acneiform eruptions, milia, and ectropion. Clearance of fibrofolliculomas has been maintained at 6 months with our patient, unlike with the use of the erbium:yttrium-aluminum-garnet as reported by Gambichler et al.^6^ Our patient may require additional “touch-up” sessions in the future. To maximize the success of treatment and minimize complications, detailed preoperative and postoperative care instructions should be reviewed in detail with the patient, including strict sun protection.

Recognition of the cutaneous manifestations and accurate diagnosis of BHDS are important given the ominous underlying associated systemic conditions. Although associated fibrofolliculomas are benign, they can have an important psychologic effect on patients with BHDS. We present a case of fibrofolliculomas in a patient with BHDS being successfully treated with 2 sessions of ablative CO_2_ laser therapy with a 92% reduction in lesion count. Given the efficacy and patient tolerability, nonfractionated ablative CO_2_ laser should be considered for the treatment of fibrofolliculomas in BHDS as well as for other benign cutaneous growths.

## Conflicts of interest

None disclosed.
